# Survival of the Fittest: Increased Stimulus Competition During Encoding Results in Fewer but More Robust Memory Traces

**DOI:** 10.3389/fpsyg.2019.00021

**Published:** 2019-01-22

**Authors:** Oliver Baumann, Eloise Crawshaw, Jessica McFadyen

**Affiliations:** ^1^Queensland Brain Institute, The University of Queensland, Saint Lucia, QLD, Australia; ^2^School of Psychology and Interdisciplinary Centre for the Artificial Mind, Bond University, Gold Coast, QLD, Australia

**Keywords:** interference, decay, forgetting, visual memory, long-term memory

## Abstract

Forgetting can be accounted for by time-indexed decay as well as competition-based interference processes. Although conventionally seen as competing theories of forgetting processes, Altmann and colleagues argued for a functional interaction between decay and interference. They revealed that, in short-term memory, time-based forgetting occurred at a faster rate under conditions of high proactive interference compared to conditions of low proactive interference. However, it is unknown whether interactive effects between decay-based forgetting and interference-based forgetting also exist in long-term memory. We employed a delayed memory recognition paradigm for visual indoor and outdoor scenes, measuring recognition accuracy at two time-points, immediately after learning and after 1 week, while interference was indexed by the number of images in a semantic category. We found that higher levels of interference during encoding led to a slower subsequent decay rate. In contrast to the findings in working-memory, our results suggest that a “survival of the fittest” principle applies to long-term memory processes, in which stimulus competition during encoding results in fewer, but also more robust memory traces, which decay at a slower rate. Conversely, low levels of interference during encoding allow more memory traces to form initially, which, however, subsequently decay at a faster rate. Our findings provide new insights into the mechanism of forgetting and could inform neurobiological models of forgetting.

## Introduction

Forgetting, defined as the inability to retrieve information, is a central feature of human memory. Two explanations for non-pathological memory loss have been proposed; one a time-indexed decay processes, the other involving competition-based interference. Forgetting by decay has traditionally been described as a passive gradual loss of the substrate of memory, due to disuse. However, newer models inspired by neurobiology describe it as an active process to remove obsolete memories, based on parameters such as relevance or recency (Hardt et al., 2013). In contrast, forgetting by interference is thought to be due to concurrent task-related mental activity. Previous studies indicate that memories are particularly vulnerable during two specific periods. First, newly formed memories are easily compromised by interference during or shortly after initial learning (Wixted, 2005; Dewar et al., 2012). Second, already consolidated long-term memories can be disrupted during the retrieval stage (Skaggs, 1933; Anderson and Neely, 1996). Further, interference effects during retrieval are strongest if the competing task-related mental activity involves stimulus material that is highly similar to the one to be encoded or retrieved (Konkle et al., 2010a,b).

Throughout the twentieth-century, time-based decay and competition-based interference theories of forgetting tended to be pitted against one another (e.g., Thorndike, 1913; Cason, 1924; Jenkins and Dallenbach, 1924; McGeoch, 1932; Brown, 1958; Keppel and Underwood, 1962; Waugh and Norman, 1965) and were often considered incompatible. This trend was broken by Altmann and colleagues (Altmann and Gray, 2002; Altmann and Schunn, 2012), who provided evidence not only for the co-existence of decay and interference, but also for the presence of interactive processes between them. Altmann’s studies showed that time-based forgetting occurred at a faster rate under conditions of high proactive interference compared to conditions of low proactive interference. These findings were interpreted as evidence for a functional role of time-based decay, which by reducing proactive interference would be instrumental in maintaining optimal working memory performance. Irrespective of whether this interaction serves the decluttering function outlined by Altmann and colleagues, such findings leave open the question of whether interactive effects between decay-based forgetting and interference-based forgetting also exist in long-term memory.

To answer this question we employed a delayed memory two-alternative forced choice (2AFC) recognition design for visual scenes, under high and low levels of encoding interference. We measured recognition accuracy at two time-points, immediately after learning and after 1 week, while interference was indexed by the number of images in a semantic category. The decay rate was therefore defined as the difference between immediate test performance and delayed test performance. The choice for this retention interval was based on previous studies, which indicate that forgetting due to decay is a relatively slow process. For instance, Vogt and Magnussen (2007) tested long-term memory for 400 visual scenes and observed a decay rate of approximately 16% over a period of 9 days. We chose natural scenes as stimulus material for two reasons. Firstly, humans have a remarkable capacity to remember visual scenes in long-term memory, even after only a single exposure to the original image. This allows testing memory performance for many exemplars and over extended periods of time. Secondly, it had been shown that varying the number of exemplars per scene category could effectively control interference levels during scene encoding. Konkle et al. (2010b) asked participants to encode thousands of scene images. By varying the number of exemplars presented per scene category and testing memory using exemplar-level foils they observed a 2% decrease in memory performance for each doubling of the number of studied scene exemplars per category. In contrast, performance was found to be unaffected by the addition of further single image categories.

Although using natural stimuli increases ecological validity it comes at a cost of experimental control. In other words, significant effects could be specific to the stimulus set employed and might not be generalizable. To assess stimulus generality we tested recognition memory separately for man-made indoor environments and natural outdoor environments. It had been shown previously that these two classes of natural scenes are distinctive in terms of both their semantic and visual characteristics (Vailaya et al., 1998; Oliva and Schyns, 2000; Torralba and Oliva, 2003). Any effects observed for both classes of natural scenes would therefore likely be generalizable to other examples of visual scenes.

When attempting to measure decay-based forgetting over multiple test time-points in a repeated measures design, it is possible that stimulus foils employed in the retrieval tasks could cause interference-related forgetting, which would constitute a confound. To circumvent this issue, we employed a two-group design, measuring decay-based forgetting as a between-subject variable, across the two classes of natural scenes.

In the absence of existing evidence for interactions between decay-based and interference-based forgetting processes in long-term memory, three competing hypotheses can be formulated. (1) Higher interference during encoding leads to few and fragile memory traces, which subsequently decay at a faster rate. (2) Higher interference during encoding leads to few but more robust memory traces, which decay at a slower rate. (3) Interference does not modulate the decay rate of long-term memories.

## Materials and Methods

### Participants

For the main study, eighty adults from the University of Queensland gave informed consent and were compensated for their participation with either $10 AUD or course credit. Fifty-nine full data sets (*M*_age_ = 21 years, 14 male) were collected. The remaining 21 datasets were unusable due to attrition (*n* = 6), technical errors (*n* = 8), and failure to meet the minimum performance criterion [70% correct in the low interference condition (*n* = 7)].

Twenty additional participants (*M*_age_ = 22 years, 4 male) provided pilot ratings of target and foil similarity prior to the commencement of testing, in order to ensure that target-foil pairings were not more similar in the low interference condition, compared with the high interference condition.

### Stimuli

Stimuli were images of 200 manmade, indoor scenes and 200 outdoor, natural scenes collected using Google image search and in accordance with a number of inclusion criteria. All images were taken from human eye level, in the daytime, in color, unlikely to evoke a strong emotional response (e.g., no hospitals, prisons, or great heights). Images containing watermarks, people, letters, digits or memorable symbols, distinctive colors, shapes, objects, or extreme weather patterns were either excluded, or these details were removed using Adobe Photoshop CS6. Natural outdoor images were required to be free of any obvious human influence, for example, fences, roads or boats. We manipulated interference by varying the number of images in a semantic category, following Konkle et al. (2010a) (see Tables 1, 2 for lists of high and low interference categories used). For both subsets of stimuli (manmade-indoor and natural-outdoor), low interference conditions were comprised of 50 images from 50 distinct semantic categories (giving one exemplar per category), and high interference conditions were comprised of 50 images from only 5 semantic categories (giving 10 exemplars from each category). Every image was paired with a similar “foil” picture during the 2AFC recognition test (hence the need for 400 images in total). Target-foil pairings were selected to be highly similar, based on features such as spatial distribution, texture, color, image quality, and object categories in the scene. Stimuli were 921 × 691 pixels, presented on a 21-inch monitor, with 1920 × 1080 screen resolution, using Presentation stimulus delivery software, version 16.2 (*Neurobehavioral Systems*).

**Table 1:** Semantic categories for man-made indoor environments.

**High interference** (50 images from 5 categories)			
Bedroom			
Church			
Laundry			
Lecture theatre			
Library			
**Low interference**	(50 images from 50 categories)		
Aeroplane	Conservatory	Gym	Recreation centre
Baggage claim	Dance studio	Hair salon	Sauna
Ballroom	Deli	Hallway	Sewing room
Basement car park	Dining room	Indoor pool	Stables
Bathroom	Domestic kitchen	Industrial kitchen	Staircase
Billiards room	Dormitory	Laboratory	Supermarket
Boardroom	Dressing room	Living room	Temple
Bowling alley	Elevator	Locker room	Theatre
Café	Food Hall	Mosque	Train
Cellar	Gallery	Nursery	Waiting room
Child’s bedroom	Garage	Office	Walk in wardrobe
Cinema	Greengrocer	Pantry	Workshop
	Greenhouse	Public toilets	


**Table 2:** Semantic categories for natural outdoor environments.

**High interference** (50 images from 5 categories)			
Alpine forest-summer			
Beach			
Desert			
Lake			
Sclerophyllous forest			
**Low interference** (50 images from 50 categories)			
Alpine	Ice desert	Seagrass meadow	Tidal channel
forest-snow	Kelp forest		Tidewater glacier
Arid desert	Mangroves	Sea-stack	Tombolo
Autumn forest	Marsh	Shale	Tropical rainforest
Bluff	Moorland	Snow-capped mountain	Tundra
Cactus forest	Mudflats		Volcano
Canyon	Oasis	Swamp	Volcano lake
Creek	Pebbled beach	Salt flat	Waterfall
Dragon tree	Prairie	Sand	Wave cut platform
forest	Red sand beach	dunes-coastal	Wet savannah
Dry savannah	Redwood forest	Sea arch	Woodland
Fjord	River	Sea cave	Xanthorrhoea forest
Glacier	Rocky coast	Sea cliff	
Grassland	Rolling hills	Temperate	
Heathland		rainforest	


### Stimulus Validation: Pilot Similarity Ratings

Prior to testing, we measured the similarity of target-foil stimulus pairs. This enabled us to ensure that the difficulty of discriminating target-foil pairs was equivalent for high interference and low interference pairs. Twenty participants (*M*_age_ = 22 years, 4 male) were asked to subjectively rate the similarity of each target-foil pair (200 pairs in total) on a 5-point Likert scale, ranging from *1 = most similar (almost identical) to 5 = least similar*. For both sets of stimuli, participants rated target-foil pairings in the *low interference* condition (manmade indoor stimuli: *M* = 3.07, *SD* = 0.49, 95% CI [2.85, 3.29]; natural outdoor stimuli: *M* = 2.74, *SD* = 0.59, CI [2.48, 3.00]) at comparable levels of similarity to images in the *high interference* condition (manmade indoor stimuli: *M* = 3.27, *SD* = 0.46, 95% CI [3.07, 3.47]; natural outdoor stimuli: *M* = 2.95, *SD* = 0.53, CI [2.72, 3.18]). It is important to note that target-foil similarity was actually lower in the high interference condition [manmade indoor: *t*(19) = 3.504, *p* = 0.002; natural outdoor: *t*(19) = 4.524, *p* < 0.001], which means that lower memory accuracy in the high-inference condition cannot be explained by higher target-foil similarity, but must be due the higher number of images per category.

### Procedure

**FIGURE 1:**
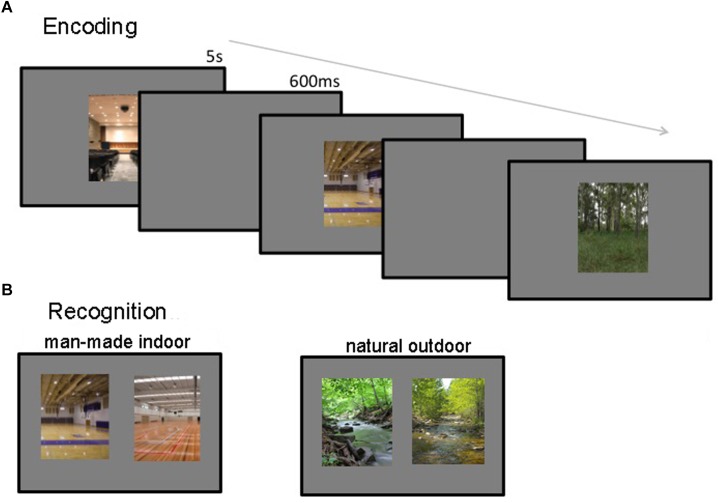
Encoding and recognition procedures. **(A)** During encoding, participants viewed 200 images for 5 s each, with a 600 ms inter-stimulus interval. **(B)** During recognition, participants viewed 100 target-foil pairs, and attempted to identify the image they had seen previously. Memory was tested immediately as well as after 1 week.

During encoding 200 images of manmade-indoor and natural-outdoor were presented randomly interleaved in the same session, appearing for 5 s, with a 600 ms inter-stimulus interval (see Figure 1A). Participants were asked to attend to the stimuli and told that they would be subsequently asked to recognize them. Participants were further shown an example trial of the recognition phase to alert them to the fact that they would have to distinguish the target from a highly similar foil image. After the encoding session the 60 participants were randomly assigned to two groups. The first group was tested immediately for manmade indoor scenes and after 1 week for natural outdoor scenes. Accordantly, the second group was tested immediately for natural outdoor scenes and after 1 week for manmade indoor scenes. This crosswise design allowed us to measure decay-based forgetting (as a between-subject variable), while avoiding potential confounds due to repeated testing of highly similar stimulus material. In addition, it allowed us to assess stimulus generality, by measuring memory accuracy for two sets of semantically and visually distinct scene images. The recognition phase consisted of 200 2AFC trials. In each trial, two images from the same scene category were presented side by side – one was a previously studied target image, and the other a distractor image that participants had not seen before (see Figure 1B). Participants were instructed to indicate which of the two scenes they had previously studied. No feedback was provided. The same set of target-foil pairs was used for all participants; however, in half of the trials, the target image was on the left side of the screen and the distractor on the right, and vice versa for the other half of the trials. Half of the trials (i.e., 100) contained images from the low-interference scene categories and the other half contained images from the high-interference scene categories. Participants proceeded at their own pace and were told to emphasize accuracy, not speed.

## Results

### Man-Made Indoor Scenes

**FIGURE 2:**
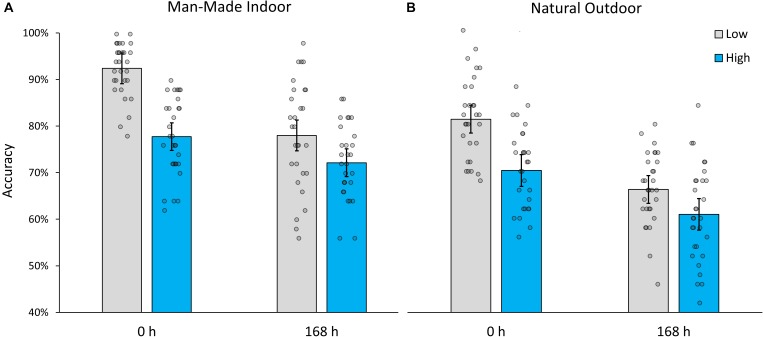
Recognition accuracy for **(A)** manmade indoor and **(B)** natural outdoor scenes encoded under low and high levels of interference, during immediate (0 h) and delayed testing (168 h). Error bars indicate 95% confidence intervals.

Accuracy for man-made indoor scenes averaged over immediate and delayed testing conditions was 85.46% (*SD* = 11.46%) for low interference images and 75.12% (*SD* = 8.51%) for high interference images, effectively replicating the finding by Konkle et al. (2010a,b). In addition, accuracy averaged over both interference conditions, decreased from 85.07% (*SD* = 10.24%) at immediate testing, to 75.12% (*SD* = 10.26%) 1 week later (168 h). A two-way mixed ANOVA indicated significant effects of interference (within-subjects factor), *F*(1,57) = 123.587, *p* < 0.001, partial-η^2^ = 0.684, and retention interval (between-subjects factor), *F*(1,57) = 22.591, *p* < 0.001, partial-η^2^ = 0.284. Importantly, their interaction was also significant, *F*(1,57) = 22.734, *p* < 0.001, partial-η^2^ = 0.285, such that memory accuracy decayed at a slower rate for images that were encoded under high levels of interference (see Figure 2A; MΔ = 5.32%, SEMΔ = 2.12%), *t*(57) = 2.507, *p* = 0.015, than under low levels (MΔ = 14.12%, SEMΔ = 2.38%), *t*(41) = 2.661, *p* < 0.001.

### Natural Outdoor Scenes

The results for natural outdoor scenes followed an analogous pattern. While overall accuracy was poorer than for indoor scenes [*t*(59) = 5.725, *p* < 0.001], the main effects and interaction remained highly significant. When averaging over immediate and delayed testing conditions, low interference images (*M* = 73.79%, *SD* = 11.13%) were better recognized than high interference images (*M* = 65.73%, *SD* = 10.45%), *F*(1,57) = 59.076, *p* < 0.001, partial-η^2^ = 0.509. Averaging over both interference conditions, accuracy also decreased significantly from 76.05% (*SD* = 10.11%) at immediate testing to 63.67% (*SD* = 9.28%) 1 week later, *F*(1,57) = 37.398, *p* < 0.001, partial-η^2^ = 0.396. Most importantly, as in Experiment 1, interference and retention interval interacted, *F*(1,57) = 6.906, *p* = 0.011, partial-η^2^ = 0.108, and the observed pattern of result (see Figure 2B) indicate once more that memory traces decay at a slower rate for images that were encoded under high levels of interference (MΔ = 9.62%, SEMΔ = 2.43%), *t*(57) = 3.969, *p* < 0.001, than under low levels (MΔ = 15.16%, SEMΔ = 2.12%), *t*(57) = 7.127, *p* < 0.001.

## Discussion

We investigated the presence of interactive effects between decay-based forgetting and interference-based forgetting in long-term memory. We found that higher levels of interference during encoding led to a slower subsequent decay rate. This indicates that competition from similar stimuli during encoding results in fewer, but also more robust memory traces, which decay at a slower rate. On the other hand, lower levels of interference during encoding allow more memory traces to form initially. Yet these memories subsequently decay at a faster rate on average than high interference memories. It is important to note that the slower decay rate for memory traces formed under high interference cannot be explained by a floor effect (i.e., no room for accuracy reduction), since performance levels in the high interference condition are significantly above the 50% baseline, for indoor as well as outdoor stimuli (one-sample *t*-test, *p* < 0.0001). Interactions between decay and interference were also conceptually replicated across two distinct stimulus sets, one containing indoor manmade scenes, and the other containing outdoor natural scenes. This suggests that the significant interaction between interference and decay is not specific to the individual stimuli chosen and is likely to be generalizable to a variety of stimulus material. Future studies should explore whether the effect generalizes to more abstract visual stimuli, such as printed or spoken words.

Our results suggest that a “survival of the fittest” principle applies to long-term memory processes, in which stimulus competition during encoding acts as “selection pressure.” This pattern is in contrast to the findings in working-memory research (Altmann and Gray, 2002; Altmann and Schunn, 2012), which indicated that higher levels of interference are associated with a faster decay rate. Given the differences in the molecular biological processes that underlie short-term and long-term memories it is not surprising that differences also exist on the behavioral level. For instance, changes in gene expression are required to convert short-term memory (STM) that lasts less than ∼1 h to long-term memory (LTM). Short-term memories are also thought to be associated with alterations in pre-existing proteins, whereas long-term memories require a protein synthesis-dependent form of synaptic plasticity (for an overview see Kandel, 2001). Future studies would, however, be necessary to further investigate the neural processes that underlie our finding.

The study by Konkle et al. (2010a) tested the effect of encoding related interference, by varying the number of items per image category during the learning phase, but only tested single items during the recognition phase (i.e., they always tested memory for the first item presented from each category). Their findings provide therefore strong evidence for retroactive interference during encoding (i.e., later items impact the encoding of the earlier item). As a sidenote, although not tested in their study it is nevertheless possible that proactive interference (i.e., earlier items impact the encoding of later items) would produce comparable results. In contrast, to the study by Konkle et al. (2010a), we tested not only one but all items per category in the high interference condition. It is therefore possible that the reduced performance we observed during recognition is not only due to encoding related interference (like in the Konkle study), but is also partially caused by interference during the recognition phase. For instance, Criss et al. (2011) reported interference effects due to items presented during the test phase, i.e., poorer performance with an increasing number of test items. The underlying idea is that each test produces a memory trace, and therefore a source of interference.

**FIGURE 3:**
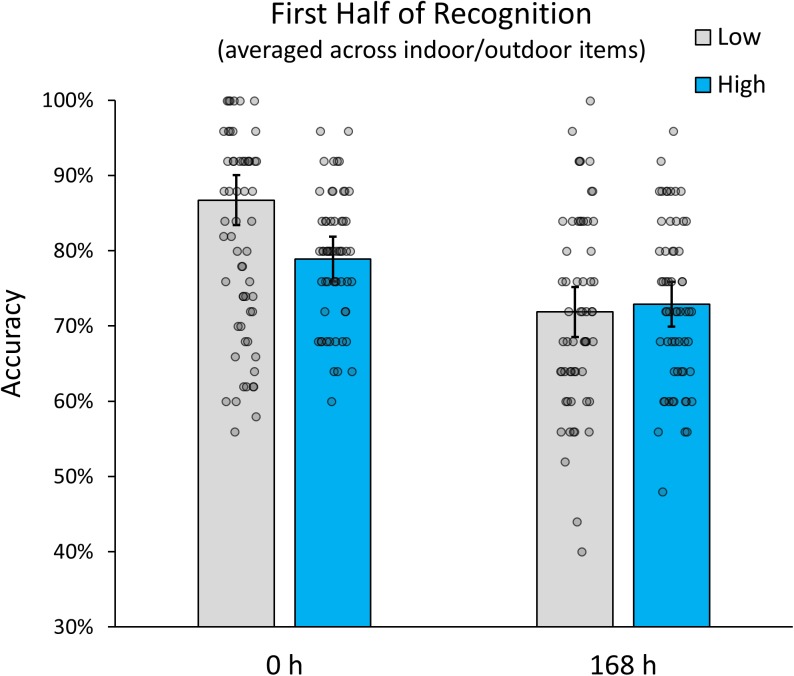
Memory accuracy for the first half of items in the recognition test (averaged over manmade indoor and natural outdoor scenes). Error bars indicate 95% confidence intervals.

To get an approximate measure of the potential impact of recognition-related interference in our study, we conducted a *post hoc* analysis that only included the first half of items tested during recognition (i.e., the first 5 items for each high interference category and the first 25 items for the low interference indoor and outdoor categories). The logic behind this partial analysis was that any improvement in memory accuracy relative to the full analysis would be indicative of a relative contribution of recognition-related interference. In addition, it would allow us to evaluate whether the observed interaction between interference and retention interval is still observable, when recognition-related interfered is reduced, while encoding-related interference is kept stable. Our analysis revealed (see Figure 3) that, while the overall effect of interference was reduced (suggesting the presence of recognition-related interference), the interaction between interference and retention interval was still significant *F*(1,57) = 19.359, *p* < 0.001. The data therefore indicate that, although recognition-related interference had an additional impact on recognition accuracy, the interactive relationship between encoding related interference and retention interval was still present. Interestingly, the additional analysis showed that, if interference during recognition is accounted for, memory performance for low and high interference items was identical at delayed testing. This seems to suggest that the initial advantage of low-interference encoding dissipates after just 1 week. It is important to note that our experiment was not designed to distinguish encoding- and recognition-related contribution to memory interference and future studies are needed to disentangle them more effectively.

Although not a theoretically motivated question of our study, we observed that memory for man-made indoor environments is better (80.07%) than for natural outdoor environments (69.78%). The difference could be due differences in low-level visual attributes of these two stimulus classes (Szummer and Picard, 1998; Oliva and Torralba, 2001). Typically, natural outdoor scenes are less structured and less distinctive than man-made indoor scenes. This greater degree of similarity could cause an increased overall level of interference compared to indoor man-made images, an effect that would explain poorer recognition performance for this image category. Alternatively, it could be that recognition performance was mediated by differences in visual expertise for the two stimulus classes. It has been shown that visual expertise leads to domain-specific increases in memory performance (Vicente and Wang, 1998; Curby et al., 2009) and it is likely that cohort of University students had on average greater exposure to man-made than natural environments.

Our study measured memory performance only for categories with 1 and 10 exemplars. Given the findings of Konkle et al. (2010a,b), which show that every doubling of the number of images per category results in a ∼2% change in recognition accuracy, we would expect the interaction effect to logarithmically increase with the number of presented items per scene category. Our study measured recognition accuracy at two time points. Another objective for future studies would be to measure retention rates at shorter as well as longer retention intervals. Although experimentally costly, a finer-grained forgetting function would provide a better indication of how the rate of forgetting of high and low interference image sets varied over time. The relationship is unlikely to be linear, given the large body of evidence indicating that forgetting over time is a curvilinear function (Wixted and Ebbesen, 1991; Wixted, 2004). In addition, measuring forgetting over longer time periods could enlighten us as to whether images encoded under high interference will eventually be better remembered than images encoded under low interference (i.e., presence of a crossover interaction), or if accuracy rates will simply converge.

A limitation of our study is that, although sources of extra-experimental variation were controlled where possible, with testing distributed throughout the day and working week to avoid time of day and time of week effects, it was not possible to control for interference that could potentially be caused by engaging in daily activities during the 1-week retention interval. Yet, this is a common factor in long-term memory research, since it would be practically impossible to isolate participants from all sources of stimulation and new learning to actually determine the potential contribution of extra-experimental interference on recognition accuracy.

In conclusion, our study provided the first evidence for the existence of interactive processes between interference-based and decay-based forgetting in long-term memory for visual scenes. Using two conceptually distinct stimulus sets, our results indicate that increased stimulus competition during encoding results in fewer but more robust memory traces, akin to a “survival of the fittest principle.” Our finding suggests that stimulus competition during encoding modulates synaptic plasticity, a hypothesis that could incorporated in computational models of forgetting, and further examined using neurophysiological techniques. Finally, our finding might have implications for educational settings, yet further investigations over longer timespans and across sensory modalities are necessary, before its relevance to real-world long-term memory maintenance can be reliably determined.

## Data Availability Statement

The data and program code are available at Open Science Framework: doi: 10.17605/OSF.IO/K7YW4. The analysis scripts use “importPresentationLog.m” written by Tobias Otto, which is available at GitHub.

## Ethics Statement

All subjects gave a written informed consent in accordance with the Declaration of Helsinki. The study was approved by the Human Research Ethics Committee of The University of Queensland.

## Author Contributions

OB and EC were responsible for study design and data acquisition. OB, EC, and JM were responsible data analysis and manuscript writing.

## Conflict of Interest Statement

The authors declare that the research was conducted in the absence of any commercial or financial relationships that could be construed as a potential conflict of interest.

## References

[B1] Altmann E. M. Gray W. D. (2002). Forgetting to remember: the functional relationship of decay and interference. *Psychol. Sci.* 13 27–33. 10.1111/1467-9280.00405 11892775

[B2] Altmann E. M. Schunn C. D. (2012). Decay versus interference: a new look at an old interaction. *Psychol. Sci.* 23 1435–1437. 10.1177/0956797612446027 23012268

[B3] Anderson M. C. Neely J. H. (1996). “Interference and inhibition in memory retrieval,” in *Memory Handbook of Perception and Cognition*, 2nd Edn, eds Bjork E. L. Bjork R. A. (San Diego, CA: Academic Press), 237–313.

[B4] Brown J. (1958). Some tests of the decay theory of immediate memory. *Q. J. Exp. Psychol.* 10 12–21. 10.1080/17470215808416249

[B5] Cason H. (1924). Criticisms of the laws of exercise and effect. *Psychol. Rev.* 31 397–417. 10.1037/h0073009 12951745

[B6] Criss A. H. Malmberg K. J. Shiffrin R. M. (2011). Output interference in recognition memory. *J. Mem. Lang.* 64 316–326.

[B7] Curby K. M. Glazek K. Gauthier I. (2009). A visual short-term memory advantage for objects of expertise. *J. Exp. Psychol. Hum. Percept. Perform.* 35 94–107. 10.1037/0096-1523.35.1.94 19170473 PMC4159943

[B8] Dewar M. Alber J. Butler C. Cowan N. Della Sala S. (2012). Brief wakeful resting boosts new memories over the long term. *Psychol. Sci.* 23 955–960. 10.1177/0956797612441220 22829465

[B9] Hardt O. Nader K. Nadel L. (2013). Decay happens: the role of active forgetting in memory. *Trends Cogn. Sci.* 17 111–120. 10.1016/j.tics.2013.01.001 23369831

[B10] Jenkins J. G. Dallenbach C. M. (1924). Obliviscence during sleep and waking. *Am. J. Psychol.* 35 605–612. 10.2307/1414040

[B11] Kandel E. R. (2001). The molecular biology of memory storage: a dialogue between genes and synapses. *Science* 294 1030–1038. 10.1126/science.1067020 11691980

[B12] Keppel G. Underwood B. J. (1962). Proactive inhibition in short-term retention of single items. *J. Verbal Learning Verbal Behav.* 1 153–161. 10.1016/S0022-5371(62)80023-1

[B13] Konkle T. Brady T. F. Alvarez G. A. Oliva A. (2010a). Conceptual distinctiveness supports detailed visual long-term memory for real-world objects. *J. Exp. Psychol. Gen.* 139 558–578. 10.1037/a0019165 20677899 PMC3398125

[B14] Konkle T. Brady T. F. Alvarez G. A. Oliva A. (2010b). Scene memory is more detailed than you think: the role of categories in visual long-term memory. *Psychol. Sci.* 21 1551–1556. 10.1177/0956797610385359 20921574 PMC3397240

[B15] McGeoch J. A. (1932). Forgetting and the law of disuse. *Psychol. Rev.* 39 352–370. 10.1037/h0069819

[B16] Oliva A. Schyns P. G. (2000). Diagnostic colors mediate scene recognition. *Cogn. Psychol.* 41 176–210. 10968925 10.1006/cogp.1999.0728

[B17] Oliva A. Torralba A. (2001). Modeling the shape of the scene: a holistic representation of the spatial envelope. *Int. J. Comput. Vis.* 42 145–175. 10.1023/A:1011139631724 16387345

[B18] Skaggs E. B. (1933). A discussion on the temporal point of interpolation and degree of retroactive inhibition. *J. Comp. Psychol.* 16 411–414. 10.1037/h0074460

[B19] Szummer M. Picard R. (1998). “Indoor-outdoor image classification. in content-based access of image and video database,” in *Proceedings of the 1998 IEEE International Workshop*, (San Jose, CA: IEEE), 42–51.

[B20] Thorndike E. (1913). *The Psychology of Learning.* New York, NY: Teachers College, Columbia University.

[B21] Torralba A. Oliva A. (2003). Statistics of natural image categories. *Network* 14 391–412. 12938764

[B22] Vailaya A. Jain A. Zhang H. J. (1998). On image classification: city images vs Landscapes. *Pattern Recognit.* 31 1921–1935.

[B23] Vicente K. J. Wang J. H. (1998). An ecological theory of expertise effects in memory recall. *Psychol. Rev.* 105 33–57. 9450371 10.1037/0033-295x.105.1.33

[B24] Vogt S. Magnussen S. (2007). Long-term memory for 400 pictures on a common theme. *Exp. Psychol.* 54 298–303. 10.1027/1618-3169.54.4.298 17953150

[B25] Waugh N. Norman D. (1965). Primary memory. *Psychol. Rev.* 72 89–104. 14282677 10.1037/h0021797

[B26] Wixted J. T. (2004). The psychology and neuroscience of forgetting. *Annu. Rev. Psychol.* 55 235–269. 10.1146/annurev.psych.55.090902.141555 14744216

[B27] Wixted J. T. (2005). A theory about why we forget what we once knew. *Curr. Dir. Psychol. Sci.* 14 6–9. 10.1111/j.0963-7214.2005.00324.x 24767478

[B28] Wixted J. T. Ebbesen E. B. (1991). On the form of forgetting. *Psychol. Sci.* 2 409–415. 10.1111/j.1467-9280.1991.tb00175.x

